# Maternal outcome after abdominal packing for uncontrolled postpartum hemorrhage despite peripartum hysterectomy

**DOI:** 10.1371/journal.pone.0177092

**Published:** 2017-06-01

**Authors:** Xavier Deffieux, Marie Vinchant, Ingrid Wigniolle, François Goffinet, Loïc Sentilhes

**Affiliations:** 1 AP-HP, GHU Sud, Hôpital Antoine Béclère, Service de Gynécologie-Obstétrique et Médecine of the Reproduction, Clamart, France; 2 AP-HP, Maternité Port Royal, Université Paris Descartes, DHU Risque et grossesses, EPOPé INSERM U953, Paris, France; 3 CHU Bordeaux, Service de Gynécologie-Obstétrique, Bordeaux, France; University of Kwazulu-Natal, SOUTH AFRICA

## Abstract

**Background:**

Intra-abdominal packing is a possible option for persistent bleeding following hysterectomy for postpartum hemorrhage. However, to date, only very limited data about maternal outcome after intra-abdominal packing for surgically uncontrolled hemorrhage following hysterectomy are available. The objective of the current study was to estimate maternal outcome after intra-abdominal packing following unsuccessful peripartum hysterectomy for postpartum hemorrhage.

**Methods:**

A questionnaire was mailed to all maternity units performing more than 850 deliveries per year. Inclusion criteria were: all cases of abdominal packing performed following unsuccessful peripartum hysterectomy for postpartum hemorrhage between 2003 and 2013. The primary outcome was success of intra-abdominal packing, defined as the arrest of hemorrhage with no need of additional procedure.

**Results:**

The total number of deliveries during the study period that occurred in the 51 participating centers was 1,430,142. The centers reported a total of 718 (1 per 2000 deliveries) peripartum hysterectomies for PPH and 53 abdominal packings performed after unsuccessful peripartum hysterectomy (about 1 per 14 hysterectomies). A median of 5 [IQR 3–7] pads were used for packing. Abdominal packing was removed after a median of 39.5 hours [IQR 24–48]. The success rate of abdominal packing was 62% (33/53). Among the 20 (38%) women in whom bleeding did not stop following the use of abdominal packing, 6 required a second surgical intervention, 6 a pelvic artery embolization and the 8 other women had “only” further intensive resuscitation and pharmacological treatments. Finally, mortality rate was 24% (13/53).

**Conclusion:**

Our results suggest that abdominal packing, used for duration of 24 to 48 hours, seems to be an option as an ultimate procedure to control persistent life-threatening postpartum hemorrhage following peripartum hysterectomy.

## Introduction

Postpartum hemorrhage (PPH) remains the main cause of peripartum maternal mortality [1–3]. Uterine-sparing surgical procedures to control severe PPH include vessels ligation (uterine and/or internal iliac arteries, stepwise uterine devascularization) and uterine compression sutures (B-Lynch suture, square) and have success rates range from 60 to 75% [4]. Peripartum hysterectomy for PPH is the ultimate surgical procedure performed immediately or when conservative measures are unsuccessful [5].

When bleeding persists after hysterectomy, very limited radiological/surgical options remain possible. Intra-abdominal packing consists in applying laparotomy pads -sterile abdominal pads- directly over the bleeding sites. The abdomen is closed under tension to maintain pressure on the packs, which are removed later (reoperation is required). This technique has been described for damage control surgery and for complex hepatic, thoracic or cancer surgery [6–13], and is associated with a decrease in mortality and morbidity in women presenting with uncontrolled intra-abdominal bleeding [7–9]. It is supposed to prevent worsening of the disseminated intravascular coagulopathy (DIC) and acidosis that usually accompany massive bleeding until they can be corrected by resuscitation measures (red blood cells, fresh frozen plasma, concentrated platelets, cryoprecipitate and fibrinogen, tranexamic acid, positive inotropic drugs, recombinant activated factor VII (rFVIIa)) [6–21].

To date, only very limited data about maternal outcome after intra-abdominal packing for surgically uncontrolled hemorrhage following hysterectomy is available. Because our current knowledge is mainly based on case reports and short case series [14–21], efficacy as well as frequency and type of severe adverse events associated with this approach remain unknown.

The primary purpose of this study was to estimate maternal outcome after intra-abdominal packing following unsuccessful peripartum hysterectomy for PPH. The secondary objective was to attempt to identify factors associated with an increase likelihood of failed abdominal packing.

## Materials and methods

A questionnaire was posted and emailed to all public and private maternity units performing more than 850 deliveries per year. The list of these maternity units was obtained using declarative data published in 2011 by the French Minister of Health on public and private hospitals (exhaustive administrative survey conducted by the *D*.*R*.*E*.*S*.*S*. (*Directorate for Research*, *Studies*, *Evaluation*, *and Statistics*). The questionnaire accompanied by a letter stating the goal of the study included items concerning the center (number of deliveries, number of hysterectomies for PPH, number of intra-abdominal packings in this context, care available in the center [intensive care unit, blood transfusion bank, pelvic embolization unit]), as well as items concerning each case of abdominal packing (patient’s characteristics, medical history, associated diseases, obstetrical complications, mode of delivery, data on PPH and on the procedure of intra-abdominal packing [technique, number of packs placed, time packs left in place]). Outcomes were also recorded (success, death, complications).

Among the 380 maternity units with more than 850 deliveries per year, 51 (13%) returned the questionnaire.

All participating hospitals collect data prospectively every day and record it in a computerized database, which may differ from one hospital to another. Midwives and residents enter data during hospitalization, immediately after delivery, and later for subsequent events. The hospitals searched their database for 2003–2013 using the following key words to identify cases: postpartum hemorrhage, peripartum hysterectomy, embolization, placenta previa, placenta accreta, placenta percreta and packing [22]. The hospitals then retrieved the paper files for each woman. An independent local investigator carefully examined the clinical notes from all files to exclude cases that met the exclusion criteria and to collect data about the women’s characteristics, pregnancy’s complications, type of delivery, modalities of PPH’s treatment including blood transfusion, surgery, embolization, intra-abdominal packing and immediate complications, and outcome.

Inclusion criteria were as follows: all consecutive cases of intra-abdominal packing performed following unsuccessful peripartum hysterectomy for PPH between 2003 and 2013. Exclusion criteria were packing of the uterine cavity, intra-abdominal packing without hysterectomy and intra-abdominal packing after hysterectomy in other context of PPH.

The primary outcome was success of intra-abdominal packing, defined as the arrest of hemorrhage with no need of any invasive procedures to control bleeding such as reoperation or embolization.

We also assessed severe maternal morbidity and complication rate following packing procedure. Severe maternal morbidity was defined as any of the following: abdominal compartment syndrome, sepsis (positive blood culture), septic shock (positive blood culture and required vasopressors to reverse sepsis-induced hypotension), peritonitis, fistula, injury to adjacent organs, acute pulmonary edema, acute renal failure, deep vein thrombophlebitis or pulmonary embolism, or maternal death [22].

Descriptive characteristics were calculated for the variables of interest. Statistical analysis included the chi-squared test and Fisher’s test for categorical variables, when the conditions of application were met, and Student’s t test or the Wilcoxon test was used for quantitative variables, according to the normality of distributions and was conducted with R software program (Lucent Technologies; http://www.r-project.org/).

This research conformed to the laws and regulations of France where the research was conducted and to generally accepted scientific principles and medical research ethical standards, and received a national Institutional Review Board (IRB) approval CEROG-OBST-2013-02-02 from the *Comité d’Ethique de la Recherche en Obstétrique et Gynécologie* (CEROG). Participant consent: 1) consent was informed; 2)where possible, written informed consent was obtained from the women. However, the mortality rate was high in this series (24%). Furthermore, since the series concerned a 10 years period, recall failed for many women.

## Results

**Fig 1:**
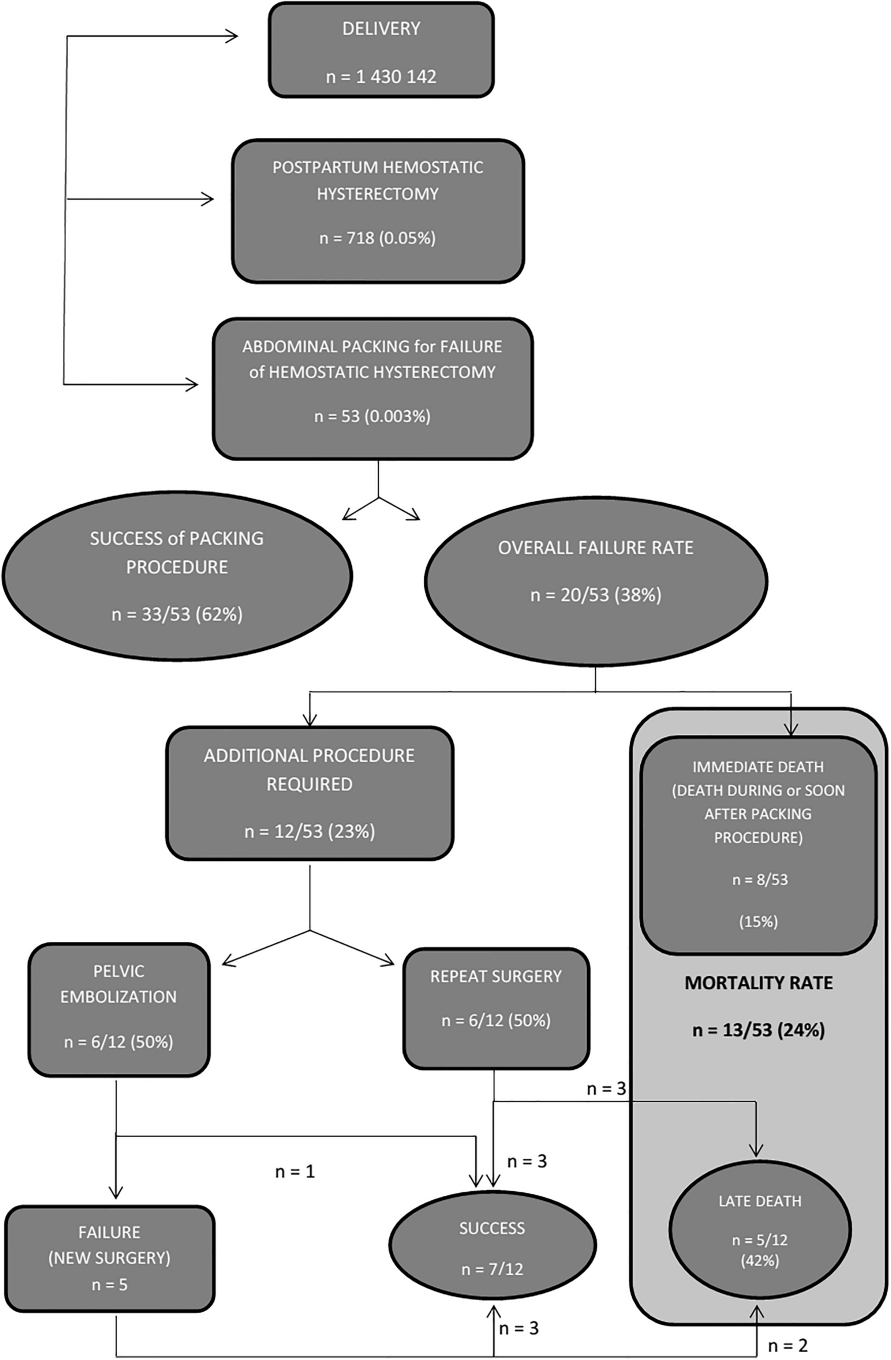
Outcomes of intra-abdominal packing for unsuccessful peripartum hysterectomy for postpartum hemorrhage.

The total number of deliveries during the study period that occurred in the 51 participating centers was 1,430,142 (Fig 1). Among the 51 participating centers, (49%) were university (tertiary care) centers, 17 (33%) were non-university public centers and 9 (18%) were private centers. The centers reported a total of 718 peripartum hysterectomies for PPH and 53 cases of abdominal packings performed after unsuccessful peripartum hysterectomy in a PPH context (Fig 1). The rate of total peripartum hysterectomy was 56% (30/53).

Twenty-five of the 51 participating centers (49%) reported to have used intra-abdominal packing at least once. Fifteen of these 25 (60%) centers reported 1 case, 4 (16%) reported 2 cases, 4 (16%) reported 3 cases and 2 (8%) reported respectively 5 and 7 cases. Participating centers’ characteristics did not differ between those that they have used at least once intra-abdominal packing and the other centers (Table 1).

**Table 1:** The participating centers’ characteristics according to they have or not used at least once intra-abdominal packing.

	Centers that reported at least one case of abdominal packing (n = 25)	Centers that did not report any case of abdominal packing (n = 26)	p
**Median [IQR] number of hysterectomies for PPH over a 10-year period**	13.5 [6.75–28.5]	8 [2–15]	.06**
**Median [IQR] number of deliveries over a 10-year period**	26781 [21,724–35,000]	25171 [20,219–30,934]	.47**
**Blood transfusion bank located in the center**	24/25 (96%)	21/26 (81%)	.21*
**Maternal Intensive Care Unit located in the center**	24/25 (96%)	21/26 (81%)	.21*
**Embolization unit located in the center**	16/25 (64%)	15/26 (58%)	.86*
**Number (%) of centers in which the number of deliveries is over 3000 per year**	10/25 (40%)	7/26 (27%)	.48*
**Number (%) of university hospitals**	15/25 (60%)	10/26 (38%)	.20*

Data are n(%), or median (interquartile range).

* Chi-squared test or Fisher’s test

** Student’s t test or Wilcoxon test

Abbreviations: IQR, interquartile range; n, number; PPH, postpartum hemorrhage.

The patient’s characteristics, data on pregnancy and delivery, as well as medical and surgical measures taken before hemostatic hysterectomy are detailed in Table 2. Laparotomy pads were used for intra-abdominal packing in 45 (85%) cases, little surgical compresses in 3 (5%) cases, and Mikulicz’s drain in 5 (10%) cases. A median 5 (IQR (interquartile range) 3–7) pads were used for packing and 4 (IQR 4–4) for Mikulicz’s drain. In 48 (90%) cases, parietal closure was complete at the end of the intervention (thus necessitating a new laparotomy to remove the pads), and an abdominal drainage was used in 24 (45%) cases. Intra-abdominal packing was removed after a median of 39.5 hours (IQR 24–48). Difficulty unsticking the pads from the viscera was reported in 2 women.

**Table 2:** Data and maternal outcome for the total population and with failed and successful packing procedure.

	Total (n = 53)	Packing success (n = 33)	Packing failure (n = 20)	P
**Characteristics of the center where packing procedure was done**				
University center	37 (69%)	23 (69%)	14 (70%)	.98*
Intensive Care Unit located in the center	52 (98%)	32 (97%)	20 (100%)	.99*
Embolization unit located in the center	38 (71%)	23 (69%)	15 (75%)	.67*
Transfusion bank located in the hospital	48 (90%)	30 (90%)	18 (90%)	.99*
Number of deliveries per year				
<1500	4 (8%)	3 (9%)	1 (5%)	
1500–3000	16 (30%)	9 (27%)	7 (35%)	.82*
>3000	33 (62%)	21 (63%)	12 (60%)	
**Patients characteristics’**				
Age (years)	35 [30–38]	34 [29–38]	36 [34–38]	.33**
BMI (kg/m^2^)	26.4 [23.9–29.1]	25.6 [23–28]	26.4 [25–29]	.14**
Parity	2 [1–3]	2 [1–3]	2 [2–3.7]	.15**
ASA score = 1	47 (88%)	1 [1–1]	1 [1–1]	.09**
Term of delivery (wg)	38 [35–39.5]	38 [36–39]	38 [35–40]	.59**
**Mode of delivery**				
Spontaneous vaginal delivery	11 (21%)	7 (21%)	4 (20%)	
Operative vaginal delivery	2 (4%)	1 (3%)	1 (5%)	.99*
Cesarean section	40 (75%))	25 (76%)	15 (75%)	
**Cause(s) of PPH**				
Uterine atony	27 (51%)	21 (63%)	6 (30%)	.01*
Placenta accreta or percreta	12 (13%)	7 (21%)	5 (25%)	.74*
Amniotic fluid embolism	6 (11%)	3 (9%)	3 (15%)	.66*
Placenta praevia	12 (22%)	10 (30%)	2 (10%)	.10*
Uterine artery injury	4 (8%)	2 (6%)	2 (10%)	.62*
Abruptio placentae	3 (5%)	1 (3%)	2 (10%)	.54*
Genital tract injury	1 (2%)	0	1 (5%)	.37*
**PPH treatment before the use of intra-abdominal packing**				
Uterotonics	53 (100%)	33 (100%)	20 (100%)	-
Oxytocin	44 (83%)	30 (90%)	14 (70%)	.06*
Sulprostone	36 (68%)	25 (75%)	11 (55%)	.13*
Recombinant activated factor VII	6 (11%)	3 (9%)	3 (15%)	.66*
Uterine-sparing surgical procedures				
Intra-uterine balloon tamponade	1 (2%)	0	1 (5%)	.37*
Uterine artery embolization	5 (10%)	3 (9%)	2 (10%)	.99*
Uterine artery ligation	13 (25%)	9 (27%)	4 (20%)	.74*
Internal iliac artery ligation	19 (36%)	13 (39%)	6 (30%)	.48*
Other vessel ligation	14 (26%)	10 (30%)	4 (20%)	.52*
Uterine compression suture	11 (21%)			
Peripartum hysterectomy	53 (100%)	33 (100%)	20 (100%)	-
**Transfusions (units)**				
Total number of red blood cells	17 [13–24]	14 [12–22]	22.5 [17–36]	.01**
Total number of frozen fresh plasma	12 [7.5–18]	10 [5.5–15]	16.5[10.7–35]	.01**
Total number of platelet cells	2 [1–5]	2 [1–3]	4[2.2–6.7]	.04**
**Interval between onset of PPH and peripartum hysterectomy (min)**	120 [46–243]	135 [60–242]	100 [45–260]	.31**
**Interval between onset of PPH and packing (min)**	285 [125–648]	270 [135–360]	300 [107–637]	.94**
**Severe maternal morbidity**	28 (53%)	12 (36%)	16 (80%)	.003*
Maternal death	13 (24%)	0	13 (65%)	<.001*
Sepsis	7 (13%)	7 (21%)	0	-
Septic shock	2 (4%)	2 (6%)	0	-
Acute pulmonary edema	9 (17%)	9 (27%)	0	-
Acute respiratory distress syndrome	9 (17%)	9 (27%)	0	-
Multiple organ failure	10 (19%)	10 (30%)	0	-
Deep vein thrombophlebitis	7 (13%)	5 (15%)	1	-
Pulmonary embolism	5 (9%)	4 (12%)	1	-
Acute renal failure	9 (17%)	9 (27%)	0	-
Necrotizing fasciitis of the buttock after embolization	1	1	0	-
Urohydronephrosis requiring drainage (pelvic hematoma)	1	1	0	-
Bowel infarction	1	0	1	-
Occlusive syndrome	2 (4%)	2 (6%)	0	-
Evisceration requiring a new surgical intervention	1	1	0	-

Data are n(%), or median [interquartile range].

* Chi-squared test or Fisher’s test

** Student’s t test or Wilcoxon test

Abbreviations: ASA, American Society of Anesthesiologists); BMI, body mass index; IQR, interquartile range; n, number; PPH, postpartum hemorrhage; wg, weeks of gestation.

The success rate of abdominal packing was 62% (33/53). Among the 20 (38%) women in whom bleeding did not stop following the use of abdominal packing, 6 required a second surgical intervention, 6 a pelvic artery embolization (Fig 1) and the 8 other women had “only” further intensive resuscitation and pharmalogical treatments. Finally, 13 women died (mortality rate 24%): 8 women died in the immediate postoperative period after abdominal packing and 5 women after reoperation or another procedure (Fig 1). Concerning the 13 cases in which death occurred, the etiologies were: multiple organ failure in 11 cases, septic shock associated with multiple organ failure in one case, and septic shock associated with bowel infarction in one case.

Antibiotic therapy lasted a median 3 days (IQR 1–7.3). The median [IQR] total of blood products transfused during patient management was 17 (IQR 13–24) units of packed red blood cells, 12 [IQR: 7.5–18] units of thawed fresh plasma, and 2 (IQR 1–5) units of platelets. Intra-abdominal pressure was not measured in any woman. The median number of days spent in intensive care was 3 (IQR 2–6,5) and the median number of hours of assisted ventilation after packing was 24 (IQR 24–48).

There were no statistically significant difference between the success and failure groups for any parameter (patients’s characteristics, mode of delivery) except for PPH etiology (initial uterine atony was associated with higher rates of success of packing procedure) (see Table 2).

## Discussion

In the current case series, abdominal packing was used in about 7% of peripartum hysterectomies due to persistent uncontrolled PPH. The success rate was 62%.

The main strengths of this study include not only the number of cases (n = 53), i.e., far the largest sample size reported on this topic, but also the fact that they have been identified from 25 participating university, non-university, public or private centers.

It is difficult to compare our results with the literature because data regarding abdominal packing for PPH are very scarce with mainly 9 single successful cases reports suggesting a publication bias [13–21]. To our knowledge, there are only small cases series totalizing 32 women that have been reported in literature [16,19]. Success rate ranged from 80 to 100% in these series [16,19,21]. A recent study reported 17 patients requiring pelvic packing after emergency peripartum hysterectomy in a PPH setting. In this series, abdominal packing successfully controlled bleeding in all cases, and the incidence of febrile morbidity was higher in the packing group than in the non-packing group [21].

The actual success rate of intra-abdominal packing is very difficult to determine since this procedure is never used alone to control bleeding and of course, it is very difficult to have a “control group”. Arrest of hemorrhage after packing is likely also secondary at least in part to intensive resuscitation and pharmacological treatment that might be pursuing after the procedure. In the opposite, mortality associated to abdominal packing might likely related to the life-threatening status of the women in whom abdominal packing is attempted to stop bleeding rather than to the procedure itself. It would be also interesting to assess the respective roles of hemostatic agents (used for bleeding from traumatized areas) associated with abdominal packing.

The high mortality rate reported in the current series, may be related to several conditions including delays in hysterectomy and the inexperience of surgeons. Early (before severe coagulopathy) peripartum hysterectomy and early packing may have advantages. However, concerning delays in hysterectomy, in the current study the interval between onset of PPH and peripartum hysterectomy was 120 min and the difference between the two groups (success and failure) was not statistically different.

The rate of total peripartum hysterectomy was 56% (30/53). Even if it is hypothesized that subtotal peripartum hysterectomy decreases morbidity and mortality, and may decrease the need for packing, in the current series, nearly half of the cases of abdominal packing followed subtotal peripartum hysterectomy.

The modalities of intra-abdominal packing are potentially determinants in its success or other maternal outcomes. David Richardson et al [23] reported that the surgical technique of abdominal packing, when performed early, reduced mortality by one-third in management of hepatic trauma. They gave no information on the type and number of compresses or pads used. In our study, we did not succeed to identify factors associated with an increase likelihood of failed abdominal packing; in particular, the median number of pads that were used and the median delay of the abdominal packing procedure from the beginning of PPH, were not founded to be statistically different between the success and failure groups.

Several problems can arise during intra-abdominal packing. If the packs are insufficiently compressive, there is a risk of persistent bleeding and thus failure. If, on the other hand, the pressure applied is too great, there is an increased risk of abdominal compartment syndrome, which has been extensively described after abdominal packing [20–25] and is defined by prolonged increase in intra-abdominal pressure above 20 mmHg, with onset of organ failure requiring abdominal decompression. In our series, intra-abdominal pressure was not measured in any woman following surgery, but no woman had suggestive symptoms of abdominal compartment syndrome.

Once intra-abdominal packing is placed, a major question is how long the packs should be left in place. There is no literature consensus, but this time ranges between 7 to 120 hours in post-partum period and in 12 to 168 hours in other context, after packing [6]. In order to avoid visceral injury, infection and/or abdominal compartment syndrome, packs are generally removed between 24 to 48 hours after packing, particularly as this time is usually sufficient to apply intensive resuscitation and control bleeding. In the context of hepatic injury, Nicol et al [26] suggest that removal after 48 hours results in less recurrence of hemorrhage, and Caruso et al [27] affirm that the risk of bleeding increases when packs are removed before 36 hours. Moreover, Abikhaled et al who assessed the impact on morbidity and mortality of abdominal packing maintained beyond 72 hours in 35 women with abdominal trauma [28], showed that removal< 72 hours was associated with a statistically significant lower rate of abscess and mortality than removal beyond 72 hours. In our study, with a median time of 39.5 hours (IQR 24–48), no visceral injury, infection related to pads and abdominal compartment syndrome occurred while no difference was found for the median time of pads removal between success and failure cases, suggesting that in the obstetrics context, the optimal time may be 24 to 48 hours.

Nevertheless, several limitations of our study must be underlined. The first is its retrospective design, common for all studies that attempted to assess outcome of ultimate procedures to control life-threatening hemorrhage. Accordingly, all the flaws of retrospective analysis apply. In particular, some eligible cases may not have been detected, especially in so far as databases may differ from one hospital to another. Nevertheless, the rate of peripartum hysterectomies observed in our study was similar to those reported in French observational studies [29,30]. Second, the length of the observation period raises concerns about possible changes in management and outcome during this time, in particular concerning pharmacological treatment and resuscitation measures.

Among the 380 maternity units with more than 850 deliveries per year, 51 (13%) returned the questionnaire. The relatively low response rate with overrepresentation of university centers (tertiary care centers) and high-volume centers may have altered the results. Nevertheless, reassuringly, indirect evidence suggests that this risk of alteration is low, as the rate of peripartum hysterectomy observed in our study was similar to those reported in French observational studies.

In conclusion, our results suggest that abdominal packing, used for a duration of 24 to 48 hours, to control persistent life-threatening PPH following peripartum hysterectomy seems to be an interesting option with no severe maternal morbidity deemed likely to be related to the procedure, resulting in a benefit/harm ratio in favor of its use as an ultimate procedure.

## Supporting information

S1 Data (XLS)
